# Distinct Biological Motion Perception in Autism Spectrum Disorder: A Meta-Analysis

**DOI:** 10.1007/s10803-021-05352-7

**Published:** 2021-11-16

**Authors:** Victoria Foglia, Hasan Siddiqui, Zainab Khan, Stephanie Liang, M. D. Rutherford

**Affiliations:** Department of Psychology, Neuroscience and Behaviour, McMaster University, 1280 Main Street West, Hamilton, ON L8S 4K1 Canada

**Keywords:** Autism spectrum disorder, Biological motion perception, Point-light display, IQ

## Abstract

If neurotypical people rely on specialized perceptual mechanisms when perceiving biological motion, then one would not expect an association between task performance and IQ. However, if those with ASD recruit higher order cognitive skills when solving biological motion tasks, performance may be predicted by IQ. In a meta-analysis that included 19 articles, we found an association between biological motion perception and IQ among observers with ASD but no significant relationship among typical observers. If the task required emotion perception, then there was an even stronger association with IQ in the ASD group.

## Introduction

Atypical social-emotional development is part of the diagnostic criteria of autism spectrum disorder (ASD) (American Psychiatric Association, 2013), and it has long been understood that social perceptual anomalies are characteristic of ASD. A recent meta-analysis of 30 studies in which a group with ASD was compared to a neurotypical (NT) group viewing point-light-walker displays confirmed a reliable moderate deficit in biological motion perception, but with a great deal of heterogeneity (Federici et al., 2020). However, relative performance by itself does not reveal whether the two groups are using the same perceptual strategy in perceiving biological motion. It is possible that observers with and without ASD who are successful at solving the perceptual task are doing so using different strategies. If typically developing observers rely on specialized perceptual mechanisms to complete the task, then one would not expect an association with IQ. However, if those with ASD do not have such specialized social perceptual processes, or if these processes are underdeveloped, they may recruit higher order cognitive skills and heuristics when solving biological motion tasks. If this were the case, performance would be predicted by IQ, but only in the ASD group.

### Biological Motion Perception

In vision science, biological motion is often portrayed with a point-light walker displays. Such displays consist of a set of points of light, each of which represents major landmarks on an actor’s body such as knees, elbows, wrists, ankles, and head (Johansson, 1973). The individual dots are perceived as biological motion if displayed altogether, rather than individually or in a scrambled formation (Johansson, 1973). Point-light displays can portray gender and actions and can include information about intent and emotional state (Brownlow et al., 1997; Cutting & Kozlowski, 1977). Perception of point-light walkers is quantified as reaction time, accuracy or detection thresholds, and tasks may require the detection of the point-light walker, the perception of the direction of the walker, or the perception of an emotion or activity of the walker (Atkinson, 2009; Todorova et al., 2019).

The perception of biological motion appears to be a specialized skill of the human perceptual system (Blake & Shiffrar, 2007; Pavlova, 2012; Rutherford, 2013). Newborns as young as 2 days old demonstrate a preference for biological motion over non-biological motion (Simion et al., 2008). The perception of point-light walkers is subject to the inversion effect (Troje & Westhoff, 2006), which is taken as evidence of specialized processing in the perception of faces (Farah et al., 1995). In addition, there is evidence of a dedicated brain region that is involved in the perception of point-light-walker displays (Grossman et al., 2000; Wheaton et al., 2001). Brain damage following stroke can result in impairment that is specific to biological motion perception (Vaina & Giese, 2002) while in another case, biological motion perception can be preserved while motion perception is impaired (Vaina et al., 1990).

### Biological Motion Perception Among Individuals with ASD

There has been much interest in the relative ability of those with ASD to perceive point-light walkers, and the results are mixed. When compared to NT controls, children with ASD have shown to be less accurate at discerning biological motion from non-biological motion (Blake et al., 2003). Some have reported that those with ASD are capable of identifying the action portrayed by point-light walkers but have difficulties in perceiving emotional content (Koldewyn et al., 2010). Annaz and colleagues (2012) and Nackaerts and colleagues (2012) reported that children with ASD show diminished biological motion processing and exhibit atypical emotion recognition compared to controls, measured both in terms of accuracy and reaction time. Similarly, Wang and colleagues (2015) reported that children with ASD lack a preference for upright point-light walkers compared to scrambled point-light walkers and show less proficiency in identifying the action of point-light walkers than their NT counterparts. In contrast, Murphy and colleagues (2009) report that if observers only had to detect a point-light walker moving left or right, response times and error rates were comparable across ASD and NT groups, suggesting no deficits in biological motion processing. Saygin and colleagues (2010) reported that impairments in biological motion for individuals with ASD depended on whether emotion perception was required: emotional perception but not motion perception tasks were difficult for the ASD group. Similarly, Hubert and colleagues (2007a, 2007b) did not find differences between groups in terms of action recognition but found differences in emotion recognition, suggesting that deficits in biological motion perception could be specific to emotion perception.

### Does IQ Predict Performance on Biological Motion Perception Tasks for Those with ASD?

There is evidence that the human visual system has specialized mechanisms designed for the perception of biological motion (Rutherford, 2013). If participants perform biological motion perception tasks by relying on specialized processes in the visual system that are dedicated to such perception, then one would no more expect an association with IQ than one would expect an association between IQ and color perception, motion perception, or size constancy.

In contrast, observers with ASD are thought to have deficits in social perception. If observers with ASD complete biological motion perception tasks by relying, in part or in whole, on higher order cognition and heuristics, then one would predict an association between task performance and IQ. This association might be evident even in studies that do not report a group difference in motion perception. Koldewyn and colleagues (2010) reported a positive correlation between IQ and behavioural performance on biological motion perception tasks within the ASD group. Rutherford and Troje (2012) reported a relationship between IQ and performance on a biological motion task in individuals with ASD, but not in the control group. Jones and colleagues (2011) reported that observers with ASD who have low IQ (but not high IQ) have poor biological motion processing. These findings are consistent with the idea that typical biological motion perception in NT individuals relies on specialized mechanisms for social perception, while individuals with ASD may employ other strategies when completing biological motion perception tasks.

### Current Study

The current meta-analysis will compare correlations between these outcome measures and IQ within each group and will compare accuracy and reaction time (RT) measured during biological motion tasks across groups with and without ASD. In addition, we will compare performance on tasks involving emotional processing to tasks which require only classification of actions and movements. We will also investigate the contribution of age and gender to differences in biological motion task performance between ASD and NT individuals.

## Methods

### Literature Search and Inclusion Criteria

This meta-analysis was designed to (1) test whether the relationship between IQ and biological motion perception was different in individuals with ASD versus NT controls and (2) estimate a pooled effect size for the difference in biological motion perception task performance between these groups. Secondarily, we tested whether the presence of emotion perception in the task, participant age and participant gender were moderating variables. There was not enough data regarding race or socio-economic status to include in these analyses. The biological motion paradigms of interest were paradigms including point-light-displays/walkers. With this objective in mind a computerized search was conducted with the key words: [(autis* OR ASD OR asperger*) and (chas* OR animate motion OR biological motion OR PLD OR point-light displays OR PLW OR point-light walker)]. The asterisks were used to allow the search to find items containing different endings of the terms to which it was applied. Dissertations and theses were excluded. The search was limited to papers published in English. The databases in which the searches were conducted were selected by the authors prior to the search. The databases selected to be searched were: PsychInfo (via proquest), PubMed, and Web of Science (via the Core Collection).

The literature search was conducted on the week of October 12th, 2020, by two authors separately to ensure accuracy and consistency. Search results were identical for both authors, yielding 323 hits on PsychInfo, 334 hits on PubMed and 482 hits on Web of Science, resulting in a total of 1139 items in the initial long list. The effectiveness of the keywords and results gathered was assessed by a third author who compared results with a Google Scholar search conducted on the week of October 27th, 2020. The first 50 Google Scholar results for the keywords “ASD and Biological motion” were found among the results of our literature search on PsychInfo, PubMed, and Web of Science. Furthermore, the search results were compared with the short list obtained by Todorova et al. (2019) who previously conducted a meta-analysis on biological motion performance and ASD individuals. All relevant articles from their short list were present in our list.

### Coding

We excluded dissertations, theses, conference proceedings, or papers not published in English. These criteria were set and discussed prior to shortening the long list, and then authors worked independently to arrive at a short list. All relevant articles were imported to Zotero, a reference manager software. We used a duplicate detecting feature in Zotero, and 329 papers were flagged as duplicates between the three databases revealing 810 unique papers in our long list.

After an article passed this first evaluation, each article was examined to ensure the rest of the following inclusion criteria were met. These criteria were agreed upon before two authors completed this check independently, to avoid any bias in included articles.

1. Study must be conducted on humans.
2. Experiment must include an ASD group and a NT control group, and they must have performed biological motion perception task with a point-light display.
3. ASD group received a formal diagnosis through ADOS or clinician and this diagnosis is reported within the study.
4. Study must report IQ scores.
5. Studies including only hand motion only were excluded.

**Fig. 1:**
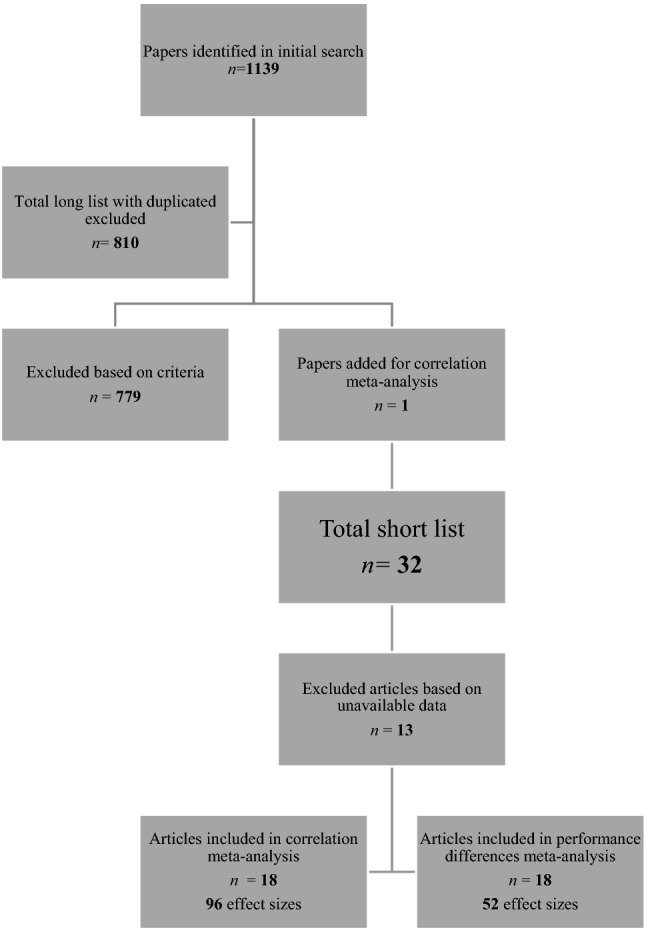
Outlining sample size of papers from initial search to data extraction

After all articles were examined and tagged, a bibliography of the included items was then exported by each reviewer and sent to the first author to check for any discrepancies and reliability. The first author calculated the prevalence and bias-adjusted kappa (PABAK) to compute the interrater reliability between the 2 authors who had finalized the article list. The obtained PABAK was 96%. After the interrater reliability was calculated all discrepancies between the 2 authors were then evaluated by the first author. The first author identified 20 discrepancies. These 20 papers were sent to an outside collaborator who had no previous access to the short list. The outside collaborator then made an independent decision to either keep or remove the articles based on the same criteria the first two reviewers used. The outside collaborator kept 9 of the 20 papers on the list, and so these were included in the short list. This left a short list of 31 articles. One additional article (Murphy et al., 2009) was added to be included in the correlational analyses, and one was removed during a data validation process by the last author, resulting in a final short list of 32 papers. See Fig. 1 to see the breakdown of the article selections moving from the long list to the short list.

### Data Extraction

All studies had the relevant data extracted independently by four of the current study’s authors to avoid bias and maximize accuracy. The third and fourth authors extracted data on group differences which was independently validated by the last author. The second and last authors independently extracted data that correlated IQ with performance. The coded variables in each article were determined prior to the data extraction. The selected 32 articles were coded for the following variables:

1. Age: Mean and standard deviation for both the ASD and NT groups.
2. Gender ratio for both participant groups.
3. Mean IQ and standard deviation for both participant groups.
4. Type of IQ measure associated with IQ score (e.g., verbal, nonverbal, full-scale, etc.)
5. Type of Paradigm: emotional vs. non emotional.
6. Performance: Mean and standard deviation for both groups.
7. Dependent Variable: Whether the paper was measuring reaction time or accuracy or threshold.
8. Effect size (standardized mean difference and/or correlation) as reported by the study.

Where accuracy and RT performance were both available, they were included as two separate entries in the analysis. There were 13 papers that did not report enough data to calculate an effect size estimate and were excluded from the final analysis. This reduced the sample size of studies included in this meta-analysis to 18 for measuring correlation between task performance and IQ and 18 papers for measuring group differences. However, multiple studies included more than one effect size, so there were 96 available effect sizes included in the analysis on the association between IQ and task performance (*n* = 60 for the ASD group). There were 52 available effect sizes included in the data analysis for group differences. See Table 1 for all papers included in the following analyses.

**Table 1:** Participant information and effect sizes (outliers indicated by*)

Author(s) year	NT sample					ASD sample					Paradigm	Measure	*g* =	95% CI’s
	*N*	Mean Age	Gender Ratio (M/F)	IQ	IQ measure	*N*	Mean Age	Gender Ratio (M/F)	IQ	IQ measure				
Actis-Grosso et al. (2015)	25	22.3	21/4	–	–	20	22.8	16/4	118.92	FSIQ	Emotional & Non-emotional	Accuracy	− 0.2004	[− 0.79155; 0.3877]
Alaerts et al. (2017)	15	23.3	15/0	114.8	FSIQ	15	21.7	1/0	107.9	FSIQ	Non-emotional	Accuracy	0.1210	[− 0.5954; 0.8375]
Atkinson (2009)	16	26.7	14/2	106.6	FSIQ	13	30.9	12/1	106.2	FSIQ	Non-emotional	Accuracy	− 0.6092	[− 1.3603; 0.1418]
Cusack et al. (2015)	18	15.54	18/0	104.79	WISC	18	16.09	18/0	103.14	WISC	Emotional	Accuracy	− 1.2624	[− 2.0735; − 0.4514]
											Non-emotional	Accuracy	− 0.2809	[− 0.9378; 0.3761]
											Non-emotional	Accuracy	− 0.2074	[− 0.9253; 0.5105]
											Non-emotional	Accuracy	0.4769	[− 0.2504; 1.2042]
											Non-emotional	Accuracy	0.2640	[− 0.3925; 0.9205]
											Non-emotional	Accuracy	0.3002	[− 0.4201; 1.0205]
											Non-emotional	Accuracy	0.0947	[− 0.6214; 0.8109]
Hubert et al., (2007a, 2007b)	19	24.3	17/2	96.4	WAIS	19	21.5	17/2	98.1		Non-emotional	Accuracy	− 1.8035*	[− 2.5702; -1.0368]*
											Non-emotional	Accuracy	− 0.8543*	[− 1.5218; -0.1869]
											Emotional	Accuracy	− 2.1189*	[− 2.9298; -1.3080]*
Jones et al. (2011)	52	15.5	49/3	88.4	FSIQ	89	15.5	81/8	85.5	FSIQ	Non-emotional	Accuracy	0.2991*	[− 0.0448; 0.6430]*
Koldewyn et al. (2011)	16	15.6	14/2	121.3	FSIQ	16	15.4	14/2	107.8	FSIQ	Non-emotional	Reaction time	− 0.1203	[− 0.6189; 0.3783]
											Non-emotional	Accuracy	− 0.5933	[− 1.1029; − 0.0837]
											Non-emotional	Accuracy	0.6957*	[0.1817; 1.2096]*
Kroger et al. (2014)	21	11.63	21/0	59.1	IQ (Raven) percent rank	17	11.9	17/0	77.6	IQ (Raven) percent rank	Non-emotional	Accuracy	0.1470	[− 0.4934; 0.7874]
											Non-emotional	Accuracy	0.1505	[− 0.4900; 0.7909]
Lindor et al. (2019)	13	9.42	7/6	111.69	FSIQ	8	10.41	5/3	112.5	FSIQ	Non-emotional	Accuracy	0.6791	[− 0.2307; 1.5888]
	13	9.42	7/6	111.69	FSIQ	10	8.96	6/4	91.2	FSIQ	Non-emotional	Accuracy	− 1.0175	[− 1.9029; − 0.1321]
Morrison et al. (2019)	95	24.17	84/11	116.28	FSIQ	103	24.28	92/11	108.9	FSIQ	Emotional	Accuracy	− 0.6125	[− 0.8979; − 0.3271]
Murphy et al. (2009)	16	26.40	13/3	56.21	FSIQ (Raven) matrices scores	16	25.56	13/3	43.73	FSIQ (Raven) matrices scores	Non-emotional	Included for correlational analysis only		
											Non-emotional	Accuracy	− 0.0639	[− 0.3428; 0.2150]
Price et al. (2012)	16	14.08	16/0	57.81	Verbal IQ	14	14.14	14/0	60.14	Verbal IQ	Non-emotional	Accuracy	− 1.1372	[− 1.9181; − 0.3563]
											Non-emotional	Accuracy	− 0.8179	[− 1.5688; − 0.0670]
											Non-emotional	Accuracy	− 0.6443	[− 1.3826; 0.0940]
van Boxtel et al. (2016)	17	13.2	13/4	112.21	FSIQ	16	14.04	12/4	101.5	FSIQ	Non-emotional	Accuracy	1.0812*	[0.3441; 1.8183]*
von der Lühe et al. (2016)	16	36.19	10/6	115.31	German multiple-choice vocabulary test	16	41.56	12/4	116.88	German multiple-choice vocabulary test	Non-emotional	Accuracy	0.0750	[− 0.6182; 0.7682]
											Non-emotional	Accuracy	− 0.6025	[− 1.3132; 0.1081]
Wright et al. (2016)	18	6.41	15/3	105.22	Verbal IQ	18	6.61	15/3	102	Verbal IQ	Non-emotional	Accuracy	− 0.0483	[− 0.7018; 0.6051]
Rutherford and Troje (2012)	14	31	14/0	117	FSIQ	14	29	14/0	117.06	FSIQ	Non-emotional	Accuracy	0.3974	[− 0.3518; 1.1467]
											Non-emotional	Accuracy	0.3338	[− 0.4130; 1.0806]
											Non-emotional	Accuracy	0.3332	[− 0.4136; 1.0799]
											Non-emotional	Accuracy	0.3889	[− 0.3600; 1.1378]
											Non-emotional	Accuracy	− 0.3475	[− 1.0947; 0.3998]
											Non-emotional	Accuracy	0.0000	[− 0.7408; 0.7408]
Freitag (2008)	15	18.6	13/2	112.1	FSIQ	15	18.6	13/2	101.2	FSIQ	Non-emotional	Reaction Time	3.1366*	[2.0243; 4.2490]*
Mazzoni et al. (2020)	27	8.81	14/13	110	NVIQ	17	12.33	15/2	44.87	NVIQ	Emotional	Reaction time	0.8960	[0.2582; 1.5337]
											Emotional	Reaction time	0.6012	[0.0441; 1.1584]
											Emotional	Reaction time	0.6319	[0.0735; 1.1904]
											Emotional	Reaction time	0.9679	[ 0.3251; 1.6107]
											Emotional	Reaction time	1.1320	[ 0.4765; 1.7875]
											Emotional	Reaction time	0.7091	[ 0.1470; 1.2713]
	27	8.81	14/13	100	NVIQ	25	9.88	44/1	110.16	NVIQ	Emotional	Accuracy	− 0.6835	[− 1.2444; − 0.1226]
											Emotional	Accuracy	− 0.5733	[− 1.1292; − 0.0173]
											Non-emotional	Accuracy	− 0.6859	[− 1.2469; − 0.1249]
											Emotional	Accuracy	− 1.1972*	[− 1.8582; − 0.5362]*
											Emotional	Accuracy	− 1.1982*	[− 1.8594; − 0.5371]*
											Non-emotional	Accuracy	− 1.9472*	[− 2.6888; − 1.2055]*
Karaminis et al. (2020)	19	13.93	8/11	105.58		19	14.15	13/6	104.32	FSIQ	Non-emotional	Accuracy	0.1305	[− 0.5061; 0.7672]
											Non-emotional	Accuracy	0.1720	[− 0.4652; 0.8092]
Kröger et al. (2014)	21	11.63	21/0	59.1		17	11.9	17/0	77.6	IQ (Raven) percent rank	Non-emotional	Reaction Time	0.1740	[− 0.4668; 0.8148]

## Results

Two separate meta-analyses were conducted. The first meta-analysis investigated the correlation between IQ and task performance within ASD and NT samples. The second meta-analysis estimated a standardized mean difference (Hedges’ *g*) for differences in biological motion task performance between ASD and NT groups. For the meta-analysis on mean differences, two pooled effect sizes were calculated, one for studies that measured accuracy and one for studies that measured reaction time.

### Correlations Between IQ and Biological Motion Perception

#### Analytic Strategy

We estimated a pooled effect size for the correlation between IQ and performance on biological motion tasks for ASD and NT groups separately. The ASD group included 60 total effect sizes from 18 different studies. The NT included 36 effect sizes from 13 different studies. Effect sizes were weighted using the inverse variance method (Hartung et al., 2011). All correlations were adjusted such that positive *r* values were associated with better performance. In the case of reaction time where negative *r* values meant performance increasing with IQ, the sign of the *r* values were inverted.

All analyses were conducted in R version 4.0.0. To calculate the pooled effect size, we used the *metacor* command in the package *meta.* Funnel plots and publication bias were assessed using the *funnelplot* and *eggers.test* command in the *dmetar* package. Subgroup analyses were conducted using the *update.meta* command in the *dmetar* package.

#### Effect Size Estimate

Using a random-effects model, we found a significant relationship between IQ and biological motion task performance in the ASD performance [*r* = 0.16, 95% CI (0.06, 0.26) *p* = 0.003].^1^ There was significant heterogeneity in the data [*Q*(59) = 240.13, *p* < 0.001, *I*^*2*^ = 75.3%], which justified the use of a random-effects model. Due to the significant heterogeneity, we assessed for the effect of outliers. Outliers were removed from the meta-analysis using the *find.outliers* command in the *demetar* package. An effect size was deemed an outlier if the 95% CI of the effect size did not overlap with the 95% CI of the pooled effect size. Using this method, 15 outliers were identified and removed. The new pooled effect size was stronger than the original effect size [*r* = 0.27, 95% CI (0.21, 0.33), *p* < 0.001]. The heterogeneity in the data was no longer significant once outliers were removed [*Q*(44) = 48.62, *p* = 0.29, *I*^2^ = 7.5%].

**Fig. 2:**
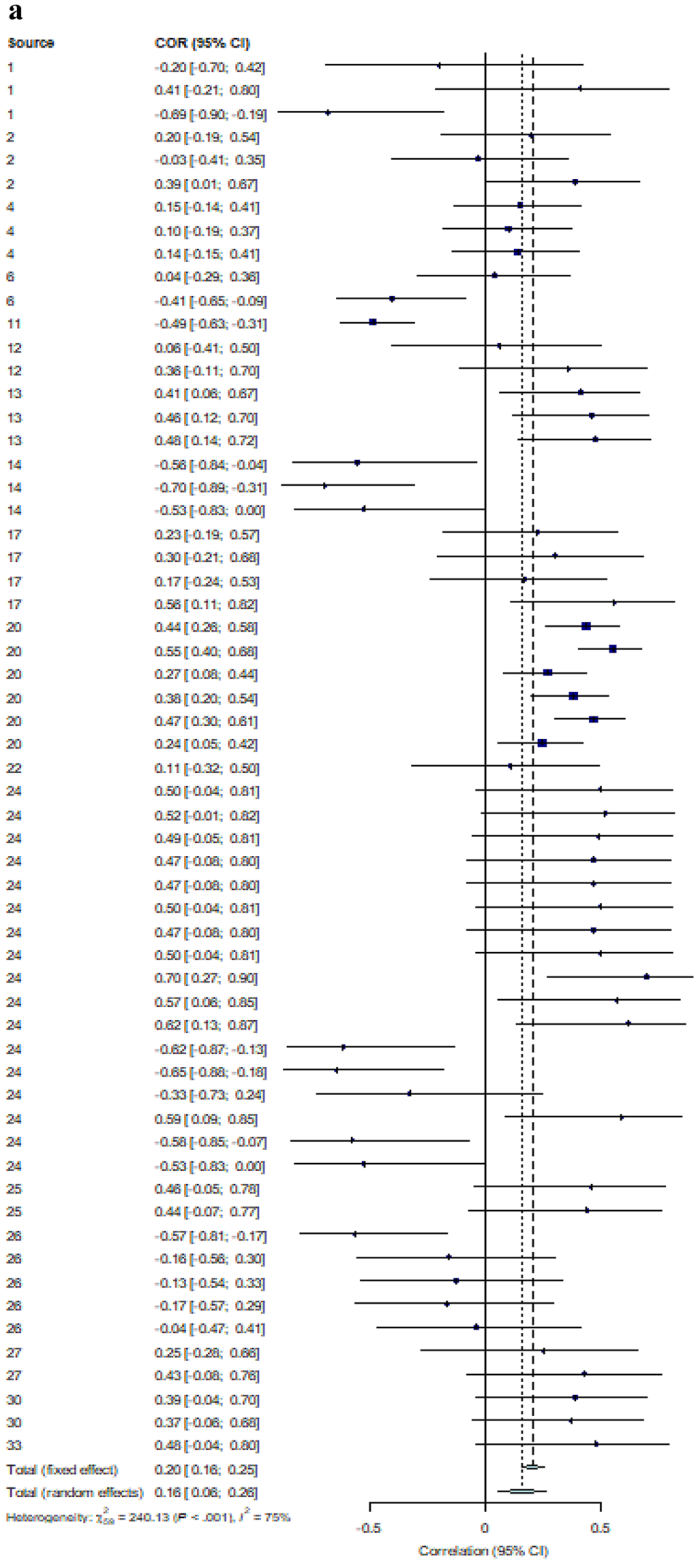

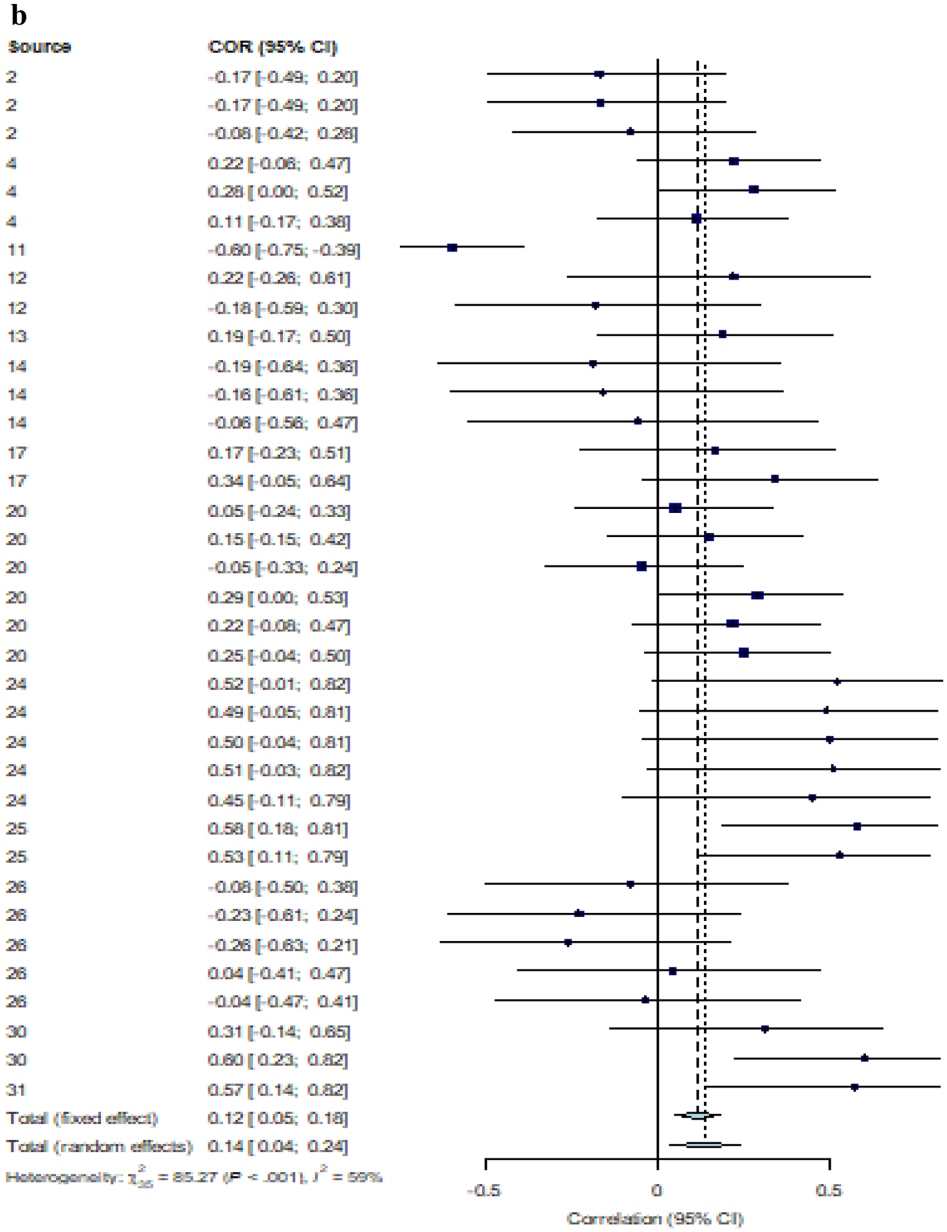
**a** Forest plot showing effect size distribution for correlations between IQ and BM task performance in the ASD group (*n* = 60). **b** Forest plot showing effect size distribution for correlations between IQ and BM task performance in the NT group (*n* = 36)

A separate random-effects model was conducted to assess the relationship between IQ and biological motion task performance in the NT group. A significant pooled correlation was found [*r* = 0.14, 95% CI (0.04, 0.24), *p* = 0.009].^2^ Similar to the ASD data, there was significant heterogeneity in our sample of effect sizes [*Q*(35) = 85.27, *p* < 0.001, *I*^2^ = 59.0%]. Again, we assessed the effect of outliers using the same method as with the ASD data. Only one outlier was identified and removed from the data. Similar to the ASD data, the new effect size was stronger than the original [*r* = 0.17, 95% CI (0.08, 0.25), *p* < 0.001]. There was still significant heterogeneity in the data, but it was reduced once the outlier was removed [*Q*(34) = 51.23, *p* = 0.03, *I*^2^ = 33.6%]. See Fig. 2a and b for a forest plot of all correlations between IQ and effect size in the ASD and NT group, respectively. Forest plots were created using the *forest* command in the *metafor* package in R 4.0.0.

#### Publication Bias

**Fig. 3:**
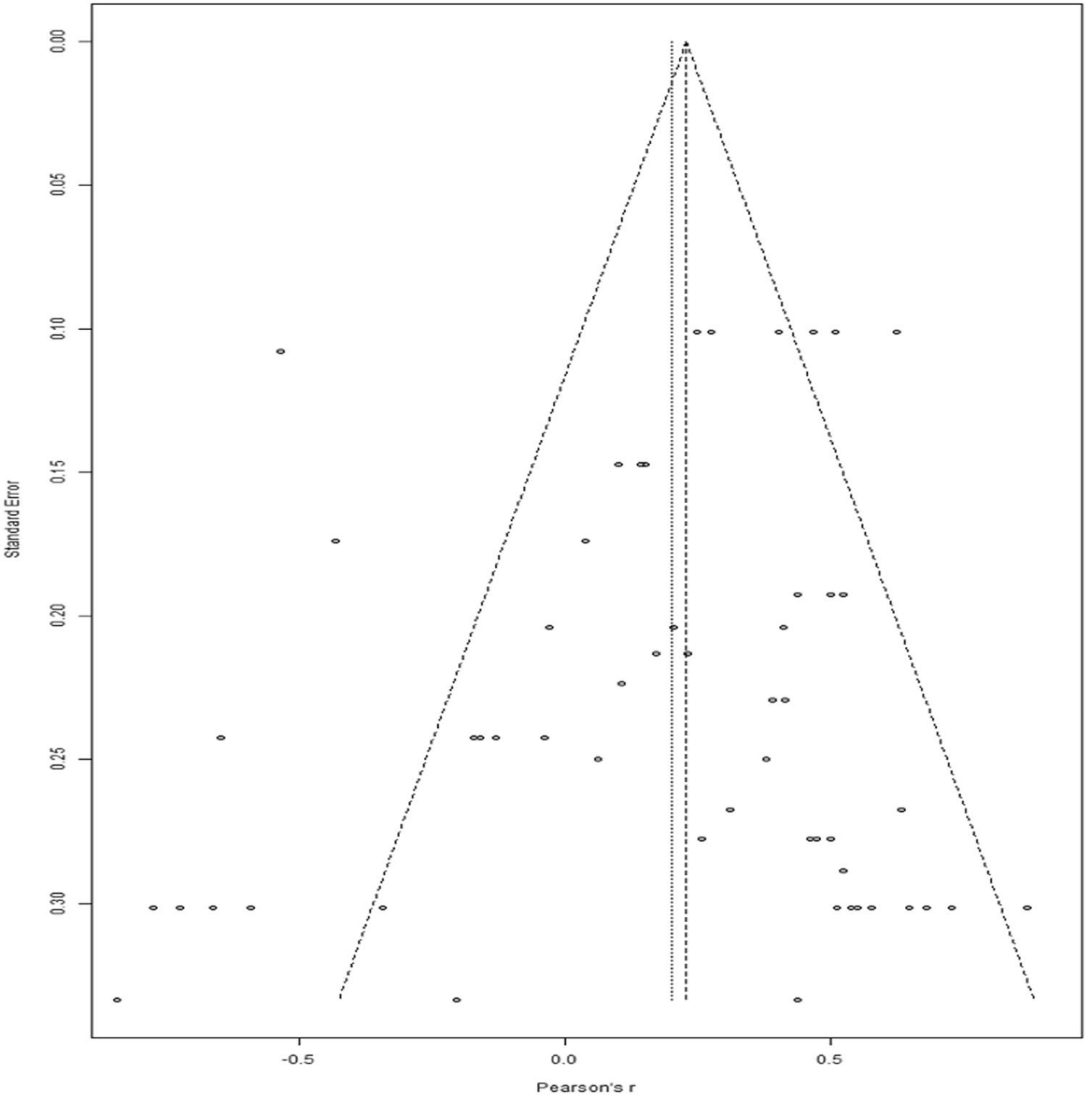
Funnel plot displays the effect sizes in relation to their standard error for the relationship between IQ and BM task performance in the ASD group. The pooled effect size from the random effect analysis is indicated by the vertical line. The x-axis represents Pearson’s r values, and the y-axis represents the standard error of the effect sizes

To gauge the presence of a publication bias we created a funnel plot for the effect sizes in the ASD group (Fig. 3). We conducted an Egger's test (Egger et al., 1997) to determine the presence of asymmetry in the funnel plot. In the presence of publication bias, the plot would show a higher concentration of studies on one side of the mean, indicating the current study includes studies with an imbalanced distribution of effect sizes. Egger’s test revealed no significant asymmetry in the funnel plot (intercept = − 0.92, *p* = 0.16). Nonetheless, when looking at the funnel plot, there did appear to be some asymmetry, with more effect sizes on the bottom left corner outside of the funnel. To ensure that publication bias was not explaining our results, we implemented Duval and Tweedie’s (2000) trim-and-fill method. However, the trim-and-fill method suggested no additions, indicating that the effect size of *r* = 0.27 is likely accurate even when considering publication bias.

**Fig. 4:**
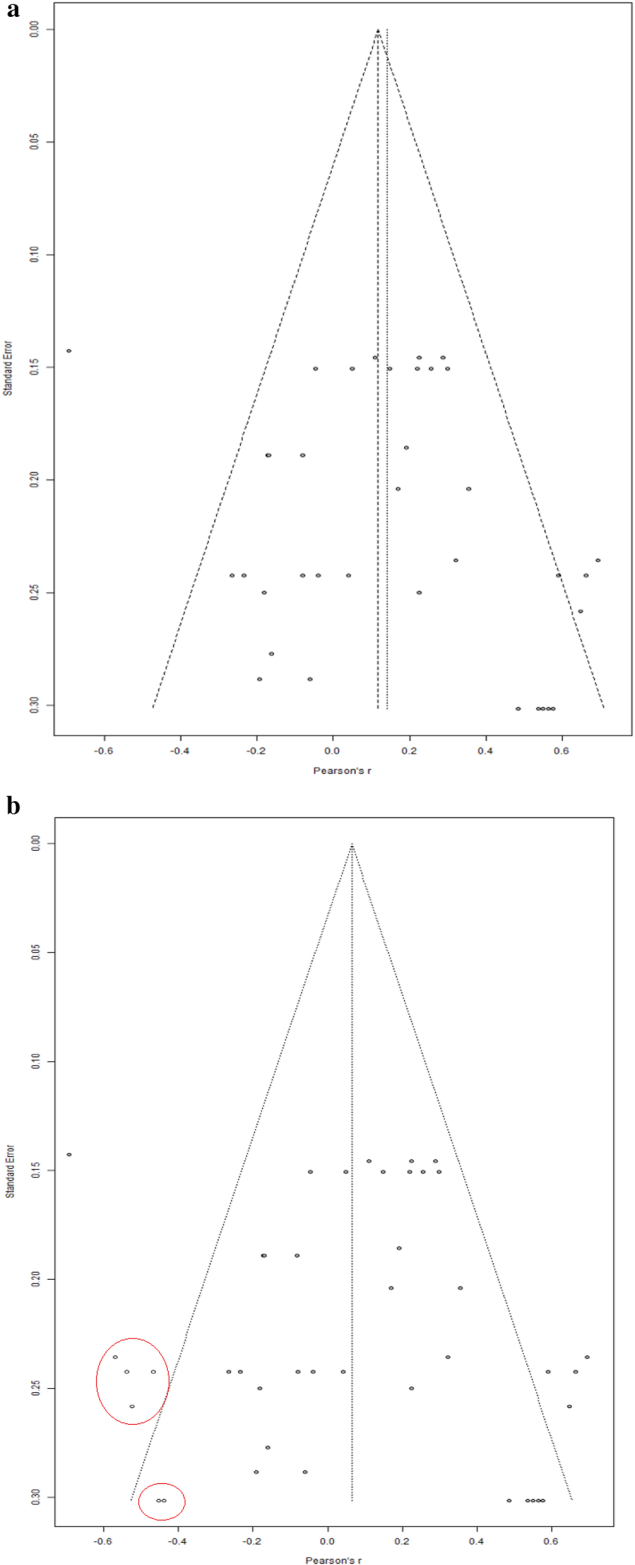
**a** Funnel plot displays the effect sizes in relation to their standard error for the relationship between IQ and BM task performance in the NT group. The pooled effect size from the random effect analysis is indicated by the vertical line. The x-axis represents Pearson’s r values, and the y-axis represents the standard error of the effect sizes. **b** Funnel plot displays the effect sizes in relation to their standard error for the relationship between IQ and BM task performance in the NT group after the addition of 6 effect sizes by the trim-and-fill method. Added effect sizes are circled in red. The pooled effect size from the random effect analysis is indicated by the vertical line. The x-axis represents Pearson’s r values, and the y-axis represents the standard error of the effect sizes

We created a second funnel plot for the NT group (Fig. 4a). We conducted an Egger’s test, and again, the Egger’s test revealed no significant asymmetry in the funnel plot (intercept = 1.51, *p* = 0.14). However, again, the funnel plot did appear asymmetric, in this case on the outside the right side of the funnel. We implemented Duval and Tweedie’s (2000) trim-and-fill method. This time, the trim-and-fill method suggested 6 additional effect sizes. Once accounting for these additional effect sizes, the new pooled effect size was much weaker, and no longer significant [*r* = 0.06, 95% CI (− 0.04, 0.17), *p* = 0.25]. A new funnel plot was created with the new effect sizes (see Fig. 4b).

#### Effect of Emotions

A secondary question of this first meta-analysis was whether the relationship between IQ and biological motion perception would be stronger for ASD participants in biological motion tasks that required emotion perception compared to non-emotional biological motion tasks, including detection of the walker, perceiving direction and perceiving an action. Prior to analysis, all effect sizes were coded as either: emotional (*n* = 17), non-emotional (*n* = 42), or mixed (*n* = 1). The 1 study labeled “mixed” was not included in this analysis, leaving 59 effect sizes. Emotionality had no significant moderating effect on the relationship between IQ and biological motion task performance [*Q*(1) = 0.51, *p* = 0.47]. However, when assessing the effect size within emotion subgroups, only the emotional subgroup pooled effect size was significant [*r* = 0.21, 95% CI (0.10, 0.32)^3^] despite it including fewer studies. The pooled effect size in the non-emotional tasks had a confidence interval that crossed zero, indicating non-significance [*r* = 0.15, 95% CI (− 0.004, 0.29)].

### Difference in Performance Between Observers With and Without ASD

#### Analytic Strategy

We estimated a pooled effect size for the differences in biological motion task performance between ASD and NT groups. We estimated two pooled effect sizes, one for studies measuring accuracy and one for studies measuring reaction time. For accuracy, 39 effect sizes were included from 17 different studies in the final meta-analysis. For reaction time, 9 effect sizes were included from 5 studies. All analyses were conducted using random-effects models.

All analyses were conducted in R version 4.0.0. To calculate the pooled effect sizes, we used the *metacont* command in the package *meta.* Funnel plots and publication bias were assessed using the *funnelplot* and *eggers.test* command in the *dmetar* package. Subgroup analyses were conducted using the *update.meta* command in the *dmetar* package.

#### Effect Size Estimates

For accuracy data, we estimated a significant pooled effect size of [*g* = − 0.30, 95% CI (− 0.50, − 0.011), *p* = 0.0025] comparing ASD groups to NT groups on accuracy-based biological motion perception tasks. There was significant heterogeneity in the model [*Q*(42) = 177.48, *p* < 0.001, *I*^2^ = 76.3%]. As such, we assessed for the effect of outliers. Outliers were removed from the meta-analysis using the *find.outliers* command in the *demetar* package. An effect size was deemed an outlier if the 95% CI of the effect size did not overlap with the 95% CI of the pooled effect size. Using this method, 8 outliers were identified and removed. The new recalculated pooled effect size was still significant [*g* = − 0.22, 95% CI (− 0.37, − 0.06), *p* = 0.0061]. There was still significant heterogeneity in the model although it was markedly reduced [*Q*(34) = 70.16, *p* = 0.0003, *I*^2^ = 51.5%].

**Fig. 5:**
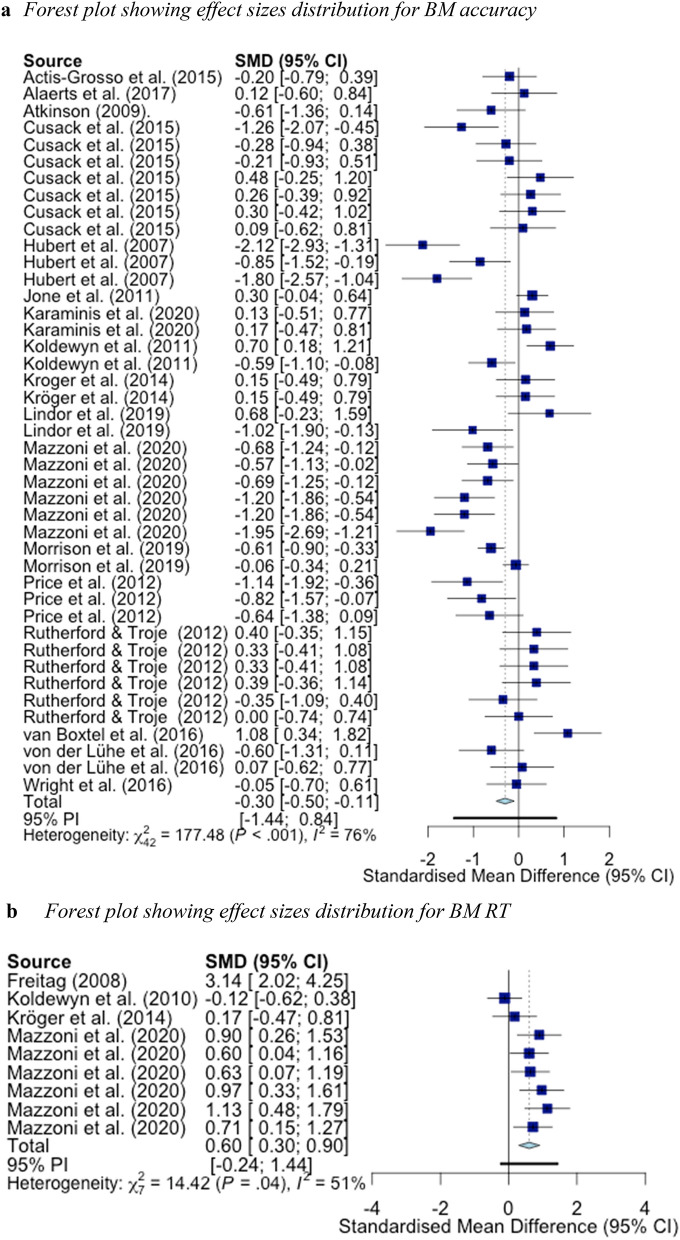
**a** Forest plot showing effect sizes distribution for BM accuracy. **b** Forest plot showing effect sizes distribution for BM RT

For RT data, we estimated a significant pooled effect size of [*g* = 0.80, 95% CI (0.37, 1.23), *p* = 0.003] comparing ASD groups to NT groups. There was significant heterogeneity in the model [*Q*(8) = 34.14, *p* ≤ 0.001, *I*^2^ = 76.6%]. As such, we assessed for the effect of outliers. Outliers were removed from the meta-analysis using the *find.outliers* command in the *demetar* package. An effect size was deemed an outlier if the 95% CI of the effect size did not overlap with the 95% CI of the pooled effect size. Using this method, 1 outlier was identified and removed. The new recalculated pooled effect size was still significant [*g* = 0.60, 95% CI (0.30, 0.90), *p* ≤ 0.001]. There was still significant heterogeneity in the model although it was markedly reduced [*Q*(7) = 14.42, *p* = 0.044, *I*^2^ = 51.5%]. See Fig. 5a and b for a forest plot of all calculated effect sizes measuring group differences. Forest plots were created using the *forest* command in the *metafor* package in R 4.0.0.

### Moderating Variables

#### Accuracy

Task stimuli, age, and gender were examined as potential moderating variables on BM accuracy. There was not enough data regarding race or socio-economic status to include in these analyses. Prior to the analysis, all effect sizes were coded as either: emotional (*n* = 7) or non-emotional (*n* = 35) for task stimuli. One other effect size was identified as both and was not included in the analyses. Using a random effects model, testing for subgroup differences between emotional and non-emotional task type performance on group differences revealed a significant moderating effect [*Q*(2) = 16.82, *p* = 0.002]. Group differences were larger on emotional tasks [*g* = − 1.01, 95% CI (− 1.37; − 0.65)] compared to non-emotional tasks [*g* = − 0.15, 95% CI (− 0.35, 0.06)]. There was no significant moderating effect of age on accuracy (*b* = 0.0047, *SE* = 0.012, *p* = 0.69) nor the gender ratio in the ASD group on accuracy performance for the ASD group (*b* = − 0.08, *SE* = 0.80, *p* = 0.92).

#### Reaction Time

The same moderating variables were assessed with respect to reaction time. Prior to the analysis, all effect sizes were coded as either emotional (*n* = 6) or non-emotional (*n* = 3). There was no significant moderating effect of the emotion on differences in RT performance [*Q*(1) = 0.05, *p* = 0.82]. There was a significant moderating effect of the gender ratio in the ASD group on accuracy performance for the ASD group (*b* = 8.69, *SE* = 4.03, *p* = 0.03), as the female proportion increased, performance was slower. There was no significant moderating effect of age on the RT of biological motion tasks (*b* = 0.16, *SE* = 0.09, *p* = 0.08).

### Publication Bias

#### Accuracy

**Fig. 6:**
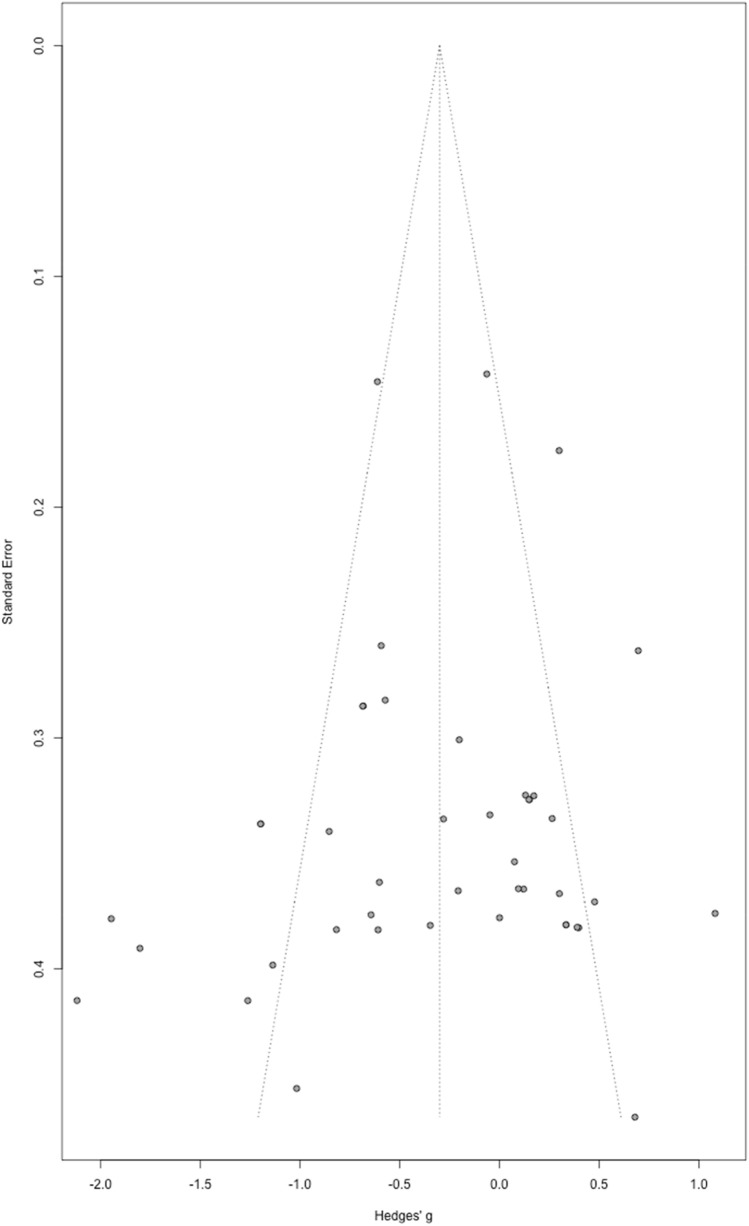
Funnel plot evaluating the possibility of a publication bias for the effect of accuracy on BM, no asymmetry in the data was observed

To evaluate the possibility of publication bias for the effect of accuracy on biological motion tasks, we created a funnel plot and performed an Egger’s test (Egger et al., 1997), using a random effects model. From the visual distribution it is evident that the effect sizes are fairly evenly distributed. The Egger’s test of the intercept did not indicate the presence of funnel plot asymmetry (intercept = − 0.76, *p* = 0.46) (see Fig. 6).

#### Reaction Time

**Fig. 7:**
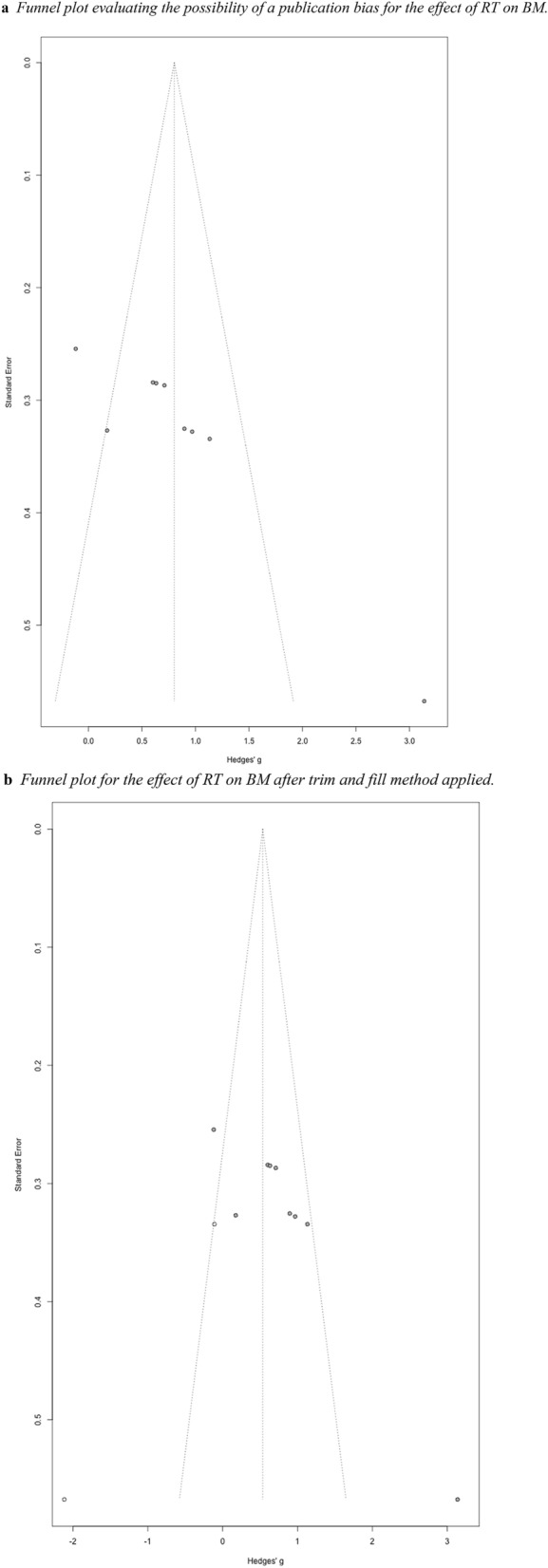
**a** Funnel plot evaluating the possibility of a publication bias for the effect of RT on BM. **b** Funnel plot for the effect of RT on BM after trim and fill method applied

While evaluating publication bias for the effect of RT on biological motion tasks, a funnel plot was created, and an Egger’s test was conducted. RT effect sizes were compared against standard error (see Fig. 7a). The Egger’s test of the intercept again indicated the presence of funnel plot asymmetry (intercept = 9.62, *p* < 0.001) (See Fig. 7a). We implemented Duval and Tweedie’s (2000) trim-and-fill method. This time, the trim-and-fill method suggested two additional effect sizes. Once accounting for these additional effect sizes, the new pooled effect size remained significant [*Q*(10) = 61.03, *p* < 0.001, *I*^2^ = 83.6%]. A new funnel plot was created with the new effect sizes (see Fig. 7b).

## Discussion

Autism spectrum disorder (ASD) is diagnosed when evidence of deficits in social communication and restricted repetitive behavior are evident in a standardized diagnostic instrument (American Psychiatric Association, 2013; Lord et al., 2012). Since social cognitive deficits in ASD may be rooted in social perception, many studies have been designed to test for group differences in performance on tasks that require social perception, including point-light walker displays of biological motion. The assumption is that differences in accuracy or reaction time are a good test of underlying psychological differences. This assumption may be problematic, since group differences in reaction time are evident in a wide variety of laboratory tasks, and group differences could, theoretically, be explained in a number of ways, including differences in attention, motivation, or an understanding of the task. Control conditions are sometimes included and are helpful in selecting among hypotheses.

A more effective approach to testing whether people with and without ASD engage in biological motion tasks differently is to measure the association between task performance and IQ and compare these associations across groups. In neurotypical groups, there is evidence suggesting that the perception of biological motion automatically engages specialized perceptual mechanisms (Grossman et al., 2000; Rutherford, 2013; Troje & Westhoff, 2006; Wheaton et al., 2001). One would not expect IQ to predict performance on a task that primarily relies on a specialized mechanism in the visual system, and we did not find a reliable association in the neurotypical group, once publication bias was corrected for. In contrast, if such a social perceptual mechanism is underdeveloped or underperforms in the ASD group, these observers might recruit other cognitive or perceptual strategies when performing the task. This would predict an association with IQ, even in the absence of group differences in accuracy or reaction time. Our meta-analysis revealed a reliable association between IQ and performance on biological motion tasks, suggesting a difference in how people with and without ASD solve such tasks.

The relationship between IQ and BM perception was significant in studies that required emotion perception, but not when only studies that did not require emotion perception were considered, despite the latter group of studies including more effect sizes. Individuals with ASD may rely on heuristics and higher-order strategies especially when required to perceive emotional content in a point-light walker display. This is not the first indication that emotionally based biological motion tasks are more difficult than non-emotional tasks among observers with ASD (Federici et al., 2020; Hubert et al., 2007a, 2007b; Saygin et al., 2010; Walsh et al., 2016). Emotion perception may be especially difficult for observers with ASD.

Across the studies we reviewed, we also found group differences in performance. We found a small but statistically significant effect size for the differences in accuracy between ASD and NT participants on biological motion tasks. Those with ASD were less accurate on biological motion perception tasks than NT controls. There was also a significant medium effect size for reaction time between the two groups, where individuals with ASD had longer RT on biological motion perception tasks than NT controls. These findings suggest that individuals with ASD may have more difficulty with biological motion processing than NT individuals. Our pooled effect sizes are similar to a previous meta-analysis which also indicated that individuals with ASD struggle with biological motion perception (Van der Hallen et al., 2019), and another recent meta-analysis reported an even larger overall effect size in differences between individuals with ASD and NT controls on biological motion tasks (Todorova et al., 2019). The current results also concur with both Annaz and colleagues (2010) and Nackaerts and colleagues (2012) who report that NT observers have higher accuracy and faster reaction times when engaged in biological motion perception.

Observers with ASD often show poorer performance compared to NT individuals on behavioral tasks that require emotion perception (Hubert et al., 2007a, 2007b; Krüger et al., 2018; Saygin et al., 2010). Because the perception of emotional states based on point-light walker displays may be a particular challenge to observers with ASD, we tested for whether the task included an emotional component predicted a contrast in performance. We found that stimuli type (i.e., emotion vs. non-emotion) moderated the effect size for group differences in accuracy. Individuals with ASD performed on par with NT individuals on non-emotional tasks, but significantly worse on emotional biological motion tasks. These results further suggest that the deficits in biological motion perception in the ASD group may be dependent on whether emotion perception is required. This result is concurrent with previous research that report differences between ASD and NT groups in biological motion emotion perception rather than biological motion action perception tasks (Federici et al., 2020; Huber et al., 2007a, 2007b; Saygin et al., 2010, Walsh et al., 2016). Similarly, Saygin and colleagues (2010) reported that deficits in biological motion perception are dependent on task type, and individuals with ASD have impaired emotion perception rather than other forms of biological motion perceptual processing. Todorova and colleagues (2019) also reported that groups with ASD exhibit poorer performance when emotional stimuli are used in biological motion processing tasks.

Our meta-analysis revealed no moderating relationship between gender ratio and age of the ASD group and effect size differences in accuracy. We also found no moderating effect of age on effect size differences in reaction time but did find a significant moderating effect of gender. The recent meta-analysis reported by Todorova and colleagues (2019) similarly did not find gender to be a moderating variable when accuracy was the dependent variable, but the current study did find a moderating effect for gender when reaction time was the dependent variable. Due to the small number of studies used to calculate the effect sizes, it is difficult to draw strong conclusions from this finding. For age, Todorova and colleagues (2019) previously reported that differences in ASD and NT performance on biological motion tasks significantly decreases with age of the ASD group: the effect is strongest among children, diminishes into adolescence, and is not robust in adults. The inconsistency between our meta-analysis and Todorova et al. (2019) may be due differences in age ranges obtained and warrants future study from other meta-analyses or experimental papers.

## Conclusion

This meta-analysis reveals significant differences between biological perception in individuals with and without ASD, and strongly suggests psychological differences in how these tasks are performed. IQ is not a robust predictor of performance on biological motion tasks among neurotypical observers but is a significant predictor of performance in samples with ASD. Our findings revealed that the ASD group had a lower accuracy on biological motion perception tasks than the NT group as well as slower RT. It may be that those with ASD rely less on specialized visual processing when perceiving biological motion and need to use other perceptual and cognitive strategies.

## Footnotes

1 We also conducted a fixed effects model to estimate the effect size, and while it was stronger, it was not significantly different as the two confidence intervals overlapped [*r* = 0.20, 95% CI (0.16, 0.25)]. Due to the heterogeneity in the data, we will henceforth only report results using the random effects model.

2 Again, we conducted a fixed-effects model to compare [*r* = 0.12, 95% CI (0.05, 0.18)]. Just like with the model using ASD data, there was no significant difference between the random- and fixed-effects models. Due to the heterogeneity of the data, we only report results using the random effects model.

3 Unfortunately, the *update.meta* function does not provide *p* values for the effect sizes within each subgroup so a confidence interval has been used instead to indicate significance.

## References

[CR1] Actis-Grosso R, Bossi F, Ricciardelli P (2015). Emotion recognition through static faces and moving bodies: A comparison between typically developed adults and individuals with high level of autistic traits. Frontiers in Psychology.

[CR3] Alaerts K, Swinnen SP, Wenderoth N (2017). Neural processing of biological motion in autism: An investigation of brain activity and effective connectivity. Scientific Reports.

[CR4] Alaerts K, Woolley DG, Steyaert J, Di Martino A, Swinnen SP, Wenderoth N (2014). Underconnectivity of the superior temporal sulcus predicts emotion recognition deficits in autism. Social Cognitive and Affective Neuroscience.

[CR5] American Psychiatric Association (2013). Diagnostic and statistical manual of mental disorders (DSM-5®).

[CR6] Annaz D, Campbell R, Coleman M, Milne E, Swettenham J (2012). Young children with autism spectrum disorder do not preferentially attend to biological motion. Journal of Autism and Developmental Disorders.

[CR7] Annaz D, Remington A, Milne E, Coleman M, Campbell R, Thomas MSC, Swettenham J (2010). Development of motion processing in children with autism. Developmental Science.

[CR8] Atkinson AP (2009). Impaired recognition of emotions from body movements is associated with elevated motion coherence thresholds in autism spectrum disorders. Neuropsychologia.

[CR9] Blake R, Shiffrar M (2007). Perception of human motion. Annual Review of Psychology.

[CR10] Blake R, Turner LM, Smoski MJ, Pozdol SL, Stone WL (2003). Visual recognition of biological motion is impaired in children with autism. Psychological Science.

[CR11] Brownlow S, Dixon AR, Egbert CA, Radcliffe RD (1997). Perception of movement and dancer characteristics from point-light displays of dance. The Psychological Record.

[CR13] Cusack JP, Williams JH, Neri P (2015). Action perception is intact in autism spectrum disorder. Journal of Neuroscience.

[CR14] Cutting JE, Kozlowski LT (1977). Recognizing friends by their walk: Gait perception without familiarity cues. Bulletin of the Psychonomic Society.

[CR15] Duval S, Tweedie R (2000). Trim and fill: A simple funnel-plot–based method of testing and adjusting for publication bias in meta-analysis. Biometrics.

[CR16] Egger M, Smith GD, Schneider M, Minder C (1997). Bias in meta-analysis detected by a simple, graphical test. BMJ.

[CR18] Farah MJ, Tanaka JW, Drain HM (1995). What causes the face inversion effect?. Journal of Experimental Psychology.

[CR19] Federici A, Parma V, Vicovaro M, Radassao L, Casartelli L, Ronconi L (2020). Anomalous perception of biological motion in autism: A conceptual review and meta-analysis. Scientific Reports.

[CR20] Freitag CM, Konrad C, Häberlen M, Kleser C, von Gontard A, Reith W, Troje NF, Krick C (2008). Perception of biological motion in autism spectrum disorders. Neuropsychologia.

[CR26] Grossman E, Donnelly M, Price R, Pickens D, Morgan V, Neighbor G, Blake R (2000). Brain areas involved in perception of biological motion. Journal of Cognitive Neuroscience.

[CR27] Hartung J, Knapp G, Sinha BK (2011). Statistical meta-analysis with applications.

[CR28] Hubert B, Wicker B, Moore DG, Monfardini E, Duverger H, Da Fonséca D, Deruelle C (2007). Brief report: Recognition of emotional and non-emotional biological motion in individuals with autistic spectrum disorders. Journal of Autism and Developmental Disorders.

[CR29] Hubert B, Wicker B, Moore DG, Monfardini E, Duverger H, Da Fonseca D, Deruelle C (2007). Brief report: Recognition of emotional and non-emotional biological motion in individuals with autistic spectrum disorders. Journal of Autism and Developmental Disorders.

[CR31] Johansson G (1973). Visual perception of biological motion and a model for its analysis. Perception & Psychophysics.

[CR32] Jones CRG, Swettenham J, Charman T, Marsden AJS, Tregay J, Baird G, Simonoff E, Happé F (2011). No evidence for a fundamental visual motion processing deficit in adolescents with autism spectrum disorders. Autism Research.

[CR33] Karaminis T, Arrighi R, Forth G, Burr D, Pellicano E (2020). Adaptation to the speed of biological motion in autism. Journal of Autism and Developmental Disorders.

[CR35] Koldewyn K, Whitney D, Rivera SM (2010). The psychophysics of visual motion and global form processing in autism. Brain.

[CR36] Koldewyn K, Whitney D, Rivera SM (2011). Neural correlates of coherent and biological motion perception in autism. Developmental Science.

[CR37] Kröger A, Bletsch A, Krick C, Siniatchkin M, Jarczok TA, Freitag CM, Bender S (2014). Visual event-related potentials to biological motion stimuli in autism spectrum disorders. Social Cognitive and Affective Neuroscience.

[CR38] Krüger B, Kaletsch M, Pilgramm S, Schwippert S-S, Hennig J, Stark R, Lis S, Gallhofer B, Sammer G, Zentgraf K, Munzert J (2018). Perceived intensity of emotional point-light displays is reduced in subjects with ASD. Journal of Autism and Developmental Disorders.

[CR40] Lindor ER, van Boxtel JJA, Rinehart NJ, Fielding J (2019). Motor difficulties are associated with impaired perception of interactive human movement in autism spectrum disorder: A pilot study. Journal of Clinical and Experimental Neuropsychology.

[CR41] Lord C, Rutter M, DiLavore P, Risi S, Gotham K, Bishop S (2012). Autism diagnostic observation schedule–2nd edition (ADOS-2).

[CR42] Mazzoni N, Landi I, Ricciardelli P, Actis-Grosso R, Venuti P (2020). Motion or emotion? Recognition of emotional bodily expressions in children with autism spectrum disorder with and without intellectual disability. Frontiers in Psychology.

[CR44] Morrison KE, Pinkham AE, Kelsven S, Ludwig K, Penn DL, Sasson NJ (2019). Psychometric evaluation of social cognitive measures for adults with autism. Autism Research.

[CR45] Murphy P, Brady N, Fitzgerald M, Troje NF (2009). No evidence for impaired perception of biological motion in adults with autistic spectrum disorders. Neuropsychologia.

[CR46] Nackaerts E, Wagemans J, Helsen W, Swinnen SP, Wenderoth N, Alaerts K (2012). Recognizing Biological motion and emotions from point-light displays in autism spectrum disorders. PLoS ONE.

[CR48] Pavlova MA (2012). Biological motion processing as a hallmark of social cognition. Cerebral Cortex.

[CR49] Price KJ, Shiffrar M, Kerns KA (2012). Movement perception and movement production in Asperger’s Syndrome. Research in Autism Spectrum Disorders.

[CR52] Rutherford MD (2013). Evidence for specialized perception of animate motion. Social Perception.

[CR53] Rutherford MD, Troje NF (2012). IQ predicts biological motion perception in autism spectrum disorders. Journal of Autism and Developmental Disorders.

[CR54] Saygin AP, Cook J, Blakemore S-J (2010). Unaffected perceptual thresholds for biological and non-biological form-from-motion perception in autism spectrum conditions. PLoS ONE.

[CR55] Simion F, Regolin L, Bulf H (2008). A predisposition for biological motion in the newborn baby. Proceedings of the National Academy of Sciences.

[CR56] Todorova GK, Hatton REM, Pollick FE (2019). Biological motion perception in autism spectrum disorder: A meta-analysis. Molecular Autism.

[CR58] Troje NF, Westhoff C (2006). The inversion effect in biological motion perception: Evidence for a “life detector”?. Current Biology.

[CR60] Vaina LM, Giese MA (2002). Biological motion: Why some motion impaired stroke patients “can” while others “can't” recognize it? A computational explanation. Journal of Vision.

[CR61] Vaina LM, Lemay M, Bienfang DC, Choi AY, Nakayama K (1990). Intact “biological motion” and “structure from motion” perception in a patient with impaired motion mechanisms: A case study. Visual Neuroscience.

[CR62] van Boxtel JJA, Dapretto M, Lu H (2016). Intact recognition, but attenuated adaptation, for biological motion in youth with autism spectrum disorder. Autism Research.

[CR64] Van der Hallen R, Manning C, Evers K, Wagemans J (2019). Global motion perception in autism spectrum disorder: A meta-analysis. Journal of Autism and Developmental Disorders.

[CR65] von der Lühe T, Manera V, Barisic I, Becchio C, Vogeley K, Schilbach L (2016). Interpersonal predictive coding, not action perception, is impaired in autism. Philosophical Transactions of the Royal Society of London.

[CR66] Walsh JA, Creighton SE, Rutherford MD (2016). Emotion perception or social cognitive complexity: What drives face processing deficits in autism spectrum disorder?. Journal of Autism and Developmental Disorders.

[CR67] Wang L-H, Chien SH-L, Hu S-F, Chen T-Y, Chen H-S (2015). Children with autism spectrum disorders are less proficient in action identification and lacking a preference for upright point-light biological motion displays. Research in Autism Spectrum Disorders.

[CR68] Wheaton KJ, Pipingas A, Silberstein RB, Puce A (2001). Human neural responses elicited to observing the actions of others. Visual Neuroscience.

[CR69] Wright K, Kelley E, Poulin-Dubois D (2016). Biological motion and the animate–inanimate distinction in children with high-functioning autism spectrum disorder. Research in Autism Spectrum Disorders.

